# Effects of *Paramisgurnus dabryanus* Density on the Growth Performance of *Pelophylax nigromaculatus* and the Soil Microbial Communities Within a Rice–Frog–Loach Integrated Aquaculture System

**DOI:** 10.3390/microorganisms13081794

**Published:** 2025-07-31

**Authors:** Chuanqi Yu, Yaping Li, Qiubai Zhou, Wenshuo Liu, Yuhong Liao, Jie Pan, Qi Chen, Haohua He, Zirui Wang

**Affiliations:** 1 College of Animal Science and Technology, Jiangxi Agricultural University, Key Laboratory of Featured Hydrobios Nutritional Physiology and Healthy Breeding, Nanchang 330045, China; yucq@jxau.edu.cn (C.Y.);; 2 School of Agricultural Sciences, Jiangxi Agricultural University, Key Laboratory of Crop Physiology, Ecology and Genetic Breeding, Ministry of Education, Nanchang 330045, China

**Keywords:** rice–frog–loach, *P. nigromaculatus*, growth performance, soil microbial communities

## Abstract

This investigation examines the influence of *P. dabryanus* density on the growth performance of *P*. *nigromaculatus* and the structural and functional dynamics of paddy soil microbial communities within a rice–frog–loach integrated aquaculture system. Field experiments were conducted with five density gradients of *P. dabryanus* (0.5, 1.0, 1.5, 2.0, and 2.5 × 10^4^ individuals/667 m^2^), designated as RFLS0.5, RFLS1.0, RFLS1.5, RFLS2.0, and RFLS2.5, respectively. Control treatments included rice monoculture (RM) and rice–frog co-culture (RFS). These findings demonstrated that as the density of loach increased, the weight gain ratio of *P. nigromaculatus* showed a unimodal pattern, reaching its peak in RFLS1. Metagenomic analysis on paddy soil revealed that the RFLS1 facilitated the enrichment of nitrogen-fixing bacteria (*Proteobacteria*), while concurrently suppressing proliferation of the potential pathogen *Pseudomonas aeruginosa* and microbial markers in metal-contaminated environments of *Usitatibacter rugosus*. Further, functional profiling indicated that RFLS1 group reached a peak activity in amino acid metabolism (14.52 ± 0.09%) and carbohydrate metabolism (14.44 ± 0.06%) and showed a higher proportion of glycosyltransferase (GT) abundance (41.93 ± 0.02%) than other groups. In summary, the optimal stocking density of *P. dabryanus* in rice–frog–loach integrated systems was determined to be 1.0 × 10^4^ individuals/667 m^2^. This density not only promotes the growth of *P. nigromaculatus* but also improves the structure of paddy soil microbial communities.

## 1. Introduction

Amid the progression of sustainable agricultural practices, rice cultivation systems are adapting to transformative demands, including diminishing reliance on chemical inputs, enhancing ecosystem service provision, and elevating agricultural product quality [1,2]. Against this backdrop, integrated rice field ecological farming systems have garnered increasing scholarly and practical attention. These systems function as a dual-production model, generating carbohydrates while supplying high-quality animal protein, and represent one of the agroecological paradigms globally [3,4]. Their capacity to enhance agricultural product diversity and safeguard nutritional security offers theoretical insights and practical pathways for advancing sustainable agricultural development. Conventional rice cultivation relies heavily on synthetic fertilizers and pesticides, a practice that induces soil compaction and diminishes microbial diversity [5,6]. Additionally, aquaculture operations experience nutrient leaching from feed into aquatic environments, promoting eutrophication [7,8,9].

To address these challenges, integrated rice–fish farming systems have emerged as an ecological agricultural technology that combines agricultural and piscine activities. By introducing fish, frogs, and other aquatic organisms into paddy fields, this system establishes a multifunctional agroecological network [10,11,12]. It ensures staple food production while generating economic returns from aquaculture. Furthermore, the feeding activities and bioturbation of fish and frogs enhance soil aeration, while their excreta provide organic fertilization, collectively ameliorating soil and water quality [13,14]. This dual ecological and economic optimization underscores the system’s potential to mitigate environmental degradation inherent in monoculture rice production and standalone aquaculture systems. The rice–frog integrated farming system represents a symbiotic agroecological model where *P. nigromaculatus* or *P. erythrus* are introduced into paddy fields, establishing a mutualistic relationship. Frogs regulate pest populations by preying on insects and weeds, while their excreta supply organic matter that increases soil-active phosphorus supplies. The paddy field, in turn, provides habitat and foraging opportunities for frogs [15]. Empirical evidence indicates that *P. nigromaculatus* stocked at approximately 15,000 individuals/hectare greatly mitigates greenhouse gas emissions from paddy fields while augmenting rice yield [16]. The introduction of *H. tigerinus* reduces pesticide dependency, enhances soil quality, and advances nutrient cycling. Additionally, this integrated system curtails nitrogen leaching and optimizes soil microbiota [17]. By elevating microbial and enzymatic activities, the system facilitates phosphorus transformation, thereby enhancing phosphorus bioavailability in the soil. This dual-function model strikes a balance between ecological sustainability and economic productivity, contributing to sustainable agricultural systems [14,18]. In organic rice–frog farming systems, ammonia-oxidizing archaea (AOA) dominate nitrification processes, whereas ammonia-oxidizing bacteria predominate in conventional paddy fields [19,20]. This microbial community divergence drastically reduces nitrate nitrogen leaching losses [21].

However, current rice–frog integrated farming practices often suffer from an imbalance between aquaculture and crop cultivation, with irrational densities escalating resource competition for space and nutrients, intensifying disease occurrence, and adversely affecting yields and economic returns. To address these challenges, the rice–frog–loach polyculture system markedly diminishes the use of chemical fertilizers and pesticides while enhancing paddy field ecological quality, thereby presenting a viable solution for advancing sustainable and eco-friendly agricultural systems. In addition, loach (*P. dabryanus*), a species of freshwater fish with considerable commercial value in East Asia, possess a remarkable resilience to oxygen-deprived conditions. This study investigated the effects of loach density on the growth performance of *P. nigromaculatus* and the structural and functional dynamics of paddy soil microbial communities within rice–frog–loach polyculture systems.

## 2. Materials and Methods

### 2.1. Polyculture and Sampling

Conducted from 1 July to 30 September 2023, in Dongxiang District, Fuzhou City, Jiangxi Province, this experiment investigated the effects of *P. dabryanus* density on the growth performance of *P. nigromaculatus* and the structural and functional dynamics of paddy soil microbial communities. Our study subjects included the late-season rice cultivar “Guangtai Youmeitezhan”, *P. nigromaculatus*, and *P. dabryanus*. The experimental design comprised seven treatment groups, each replicated three times in parallel units of 222 m^2^: rice monoculture (RM), rice–frog co-culture (RFS), and rice–frog–loach polyculture (RFLS) with five density gradients of *P. dabryanus*: 0.5, 1.0, 1.5, 2.0, and 2.5 × 10^4^ individuals/667 m^2^. The number of *P. dabryanus* per kilogram is calculated, and then weighing is used to calculate the number of individuals in the area. Our experimental paddy field was enclosed with perimeter fencing and equipped with surrounding ditches 200 cm long, 80 cm wide, and 50 cm deep. The late-season rice cultivar “Guangtai Youmeitezhan” was planted around the water channels at a spatial arrangement of 20 cm × 30 cm between plants. When water temperatures exceeded 20 °C, *P. nigromaculatus* tadpoles (5 g/individual) were introduced at a density of 1.5 × 10^4^ individuals/667 m^2^. After rice regreening, 15 days after rice transplantation, *P. dabryanus* fry (3.25 ± 0.09 g/individual) were stocked. Each parallel unit was isolated with protective netting and managed with independent drainage and irrigation systems. Throughout this experiment, paddy field management followed conventional rice cultivation practices.

Upon rice maturation, 30 individuals of *P. nigromaculatus* were randomly sampled from each replicate group and individually weighed to record body mass. Soil samples were collected using the S-type nine-point sampling method in paddy fields. Subsequently, 15 individuals of *P. nigromaculatus* were randomly selected from the initial 30, and their visceral mass, liver, and hind legs were weighed. Morphometric indices, including weight gain ratio, hepatosomatic index, viscerosomatic index, and leg-to-body ratio, were calculated based on these measurements to evaluate the growth performance and somatic composition of *P. nigromaculatus*. This study was performed in accordance with recommendations for animal care and ethics in China. Moreover, our study has been subjected to scrutiny by the Animal Experiment Ethics Committee of Jiangxi Agricultural University and has received formal sanction.

### 2.2. Soil Metagenomic Sequencing

Nucleic acid extraction from pretreated soil samples was performed with the OMEGA Soil DNA Kit (D5635-02, Omega Bio-Tek, Norcross, GA, USA). Then, extracted DNA underwent 0.8% agarose gel electrophoresis to assess molecular size and was quantified via Nanodrop analysis. Thereafter, with the help of the Illumina TruSeq Nano DNA LT Library Preparation Kit (San Diego, CA, USA), the extracted DNA was crafted into metagenome sequencing libraries with 400 bp insert sizes.

A 1-μL library aliquot was subjected to quality assessment through the Agilent High Sensitivity DNA Kit on an Agilent Bioanalyzer 2100 (Santa Clara, CA, USA). A qualified library must demand a single peak profile and be free of adapter contamination. Subsequently, the library was quantified with the Quant-iT PicoGreen dsDNA Assay Kit on a Promega QuantiFluor instrument (Madison, WI, USA). The calculated concentration of a qualified library must reach at least 2 nM. Qualified libraries were subjected to sequencing on the Illumina MiSeq platform (PE300 paired-end sequencing). Libraries, marked with non-redundant indices, were first serially diluted to 2 nM and then pooled in proportion to the required data volume. The pooled libraries were denatured into single strands via 0.1N NaOH prior to sequencing. The final concentration of the libraries was adjusted to a range of 15–18 pM, with specific adjustments made according to experimental requirements.

Raw sequence data were analyzed through QIIME2 version 2022.11, following the official protocol. Initially, the demux plugin was utilized for demultiplexing. Subsequently, the cutadapt plugin executed primer removal. Then the DADA2 plugin performed quality filtering, denoising, merging, and chimera removal. Finally, sequences were clustered at 100% similarity to generate ASVs and abundance tables. Upon the application of QIIME2, taxonomic composition and abundance data were obtained for microbial samples across six taxonomic ranks: phylum, class, order, family, genus, and species. Results were visualized via bar charts. Differences in microbial community structure between groups were assessed for significance via PERMANOVA (Adonis/PERMANOVA analysis). LEfSe analysis was employed to detect taxonomic units exhibiting statistically significant differences between experimental groups. The functionality of the non-redundant genes was performed using PICRUSt2 (phylogenetic investigation of communities by reconstruction of unobserved states), leveraging the Carbohydrate-Active enZYmes (CAZy) and Kyoto Encyclopedia of Genes and Genomes (KEGG) databases. According to the predicted metabolic profiles, principal coordinate analysis (PCoA) was conducted on functional units. Differential metabolic pathways between groups were identified with adjusted *p*-values (adjPvalues) and log fold change (logFC) thresholds. Additionally, the taxonomic composition of organisms associated with these metabolic pathways was analyzed.

### 2.3. Statistical Analysis

Data of each group were expressed as mean ± standard error (mean ± S.E.). SPSS 25.0 software was used for analyzing the data. The Kolmogorov–Smirnov and Levene’s tests were used to evaluate the normality and homogeneity of variance of data, respectively. Subsequently, the statistical differences between treatments were evaluated by one-way ANOVA and Tukey’s multi-range test. *p* < 0.05 was the level of significance.

## 3. Results

### 3.1. Effects of Loach Density on Growth Performance of *Frog*

Growth performance of frogs is presented in Table 1. The growth indices (weight gain ratio, hepatosomatic index, and viscerosomatic index) represented a unimodal pattern, peaking in the RFLS 1 group. Moreover, the weight gain ratio, hepatosomatic index, and viscerosomatic index were significantly increased in the RFLS 1 group compared with the RFS group (*p* < 0.05). Additionally, the parameter of leg-to-body ratio was significantly lower in RFLS 0.5–2.5 groups when compared to the RFS group (*p* < 0.05).

### 3.2. Impact of Loach Density on Paddy Soil Microbial Composition

**Figure 1:**
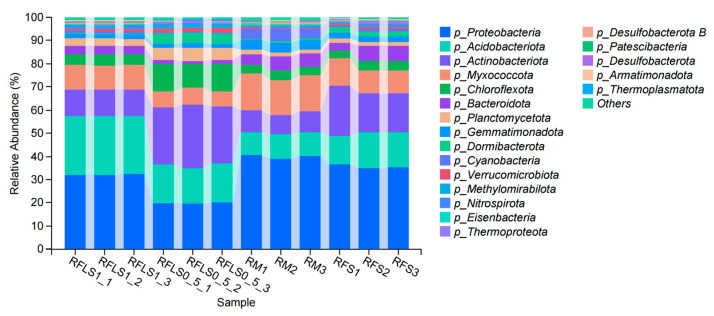
Taxonomic analysis of soil microbiota at the phylum level.

**Figure 2:**
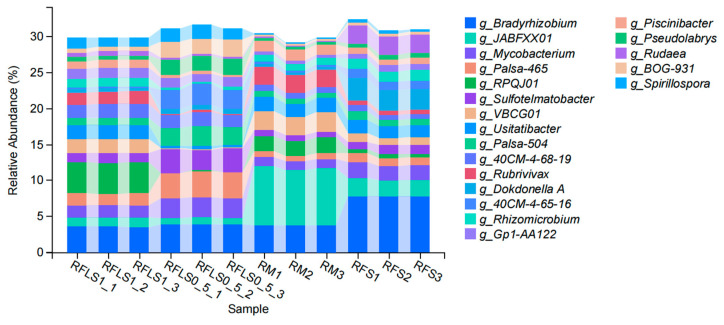
Taxonomic analysis of soil microbiota at the genus level.

**Figure 3:**
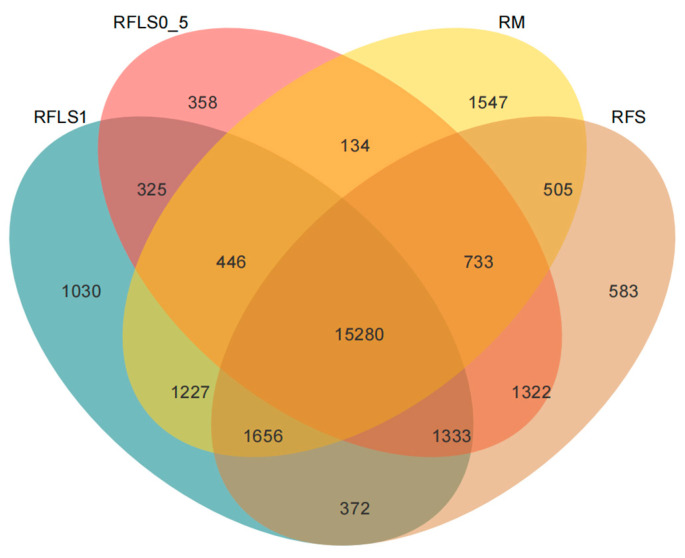
Venn diagram analysis of soil microbial species.

**Figure 4:**
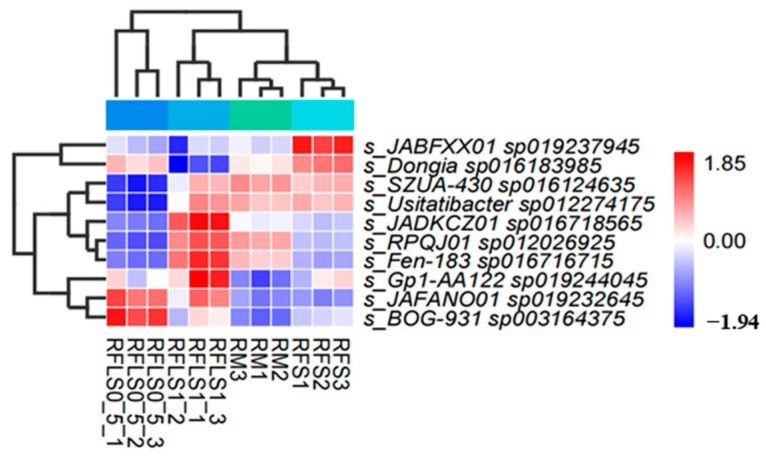
Heatmap analysis of differential soil microbial species.

As shown in Figure 1, the dominant phyla of paddy soil microbial communities represented significant variation across different treatments. The RM group was predominantly composed of *Proteobacteria*, *Myxococcota*, and *Acidobacteriota*. In the RFS group, the dominant phyla were *Proteobacteria*, *Actinobacteriota*, and *Acidobacteriota*. The RFLS 0.5 group was primarily dominated by *Actinobacteriota*, *Proteobacteria*, and *Acidobacteriota*. RFLS 1 group showed a predominance of *Proteobacteria*, *Acidobacteriota*, and *Actinobacteriota*. As shown in Figure 2, *Mycobacterium* reached peak abundance in the RFLS 1 group, while *Sulfotelmatobacter* demonstrated the highest abundance in the RFLS 0.5 group. *Bradyrhizobium* showed higher abundance in both the RFLS 1 and RFLS 0.5 groups. *Usitatibacter* achieved its highest abundance in the RFLS 1 group. Figure 3 illustrates that those four treatment groups shared 15,280 species, with unique species counts as follows: 1547 in the RM group, 583 in the RFS group, 358 in the RFLS 0.5 group, and 325 in the RFLS 1 group. As shown in Figure 4, the optimal density loach (RFLS 1) treatment significantly suppressed the abundance of *Usitatibacter rugosus* (sp012274175) compared with other groups. The highest abundance of *Pseudomonas aeruginosa* (sp016124635) was found in the rice monoculture (RM) group. Conversely, the rice monoculture (RM) treatment significantly inhibited the abundance of *Faecalibacterium prausnitzii* (sp019244045) compared with other groups. In comparison, with the RFLS 0.5 group, the abundance of *Dongia mobilis* (sp016183985) was significantly decreased in RFLS 1.

### 3.3. Impact of Loach Density on Paddy Soil Microbial Functions

**Figure 5:**
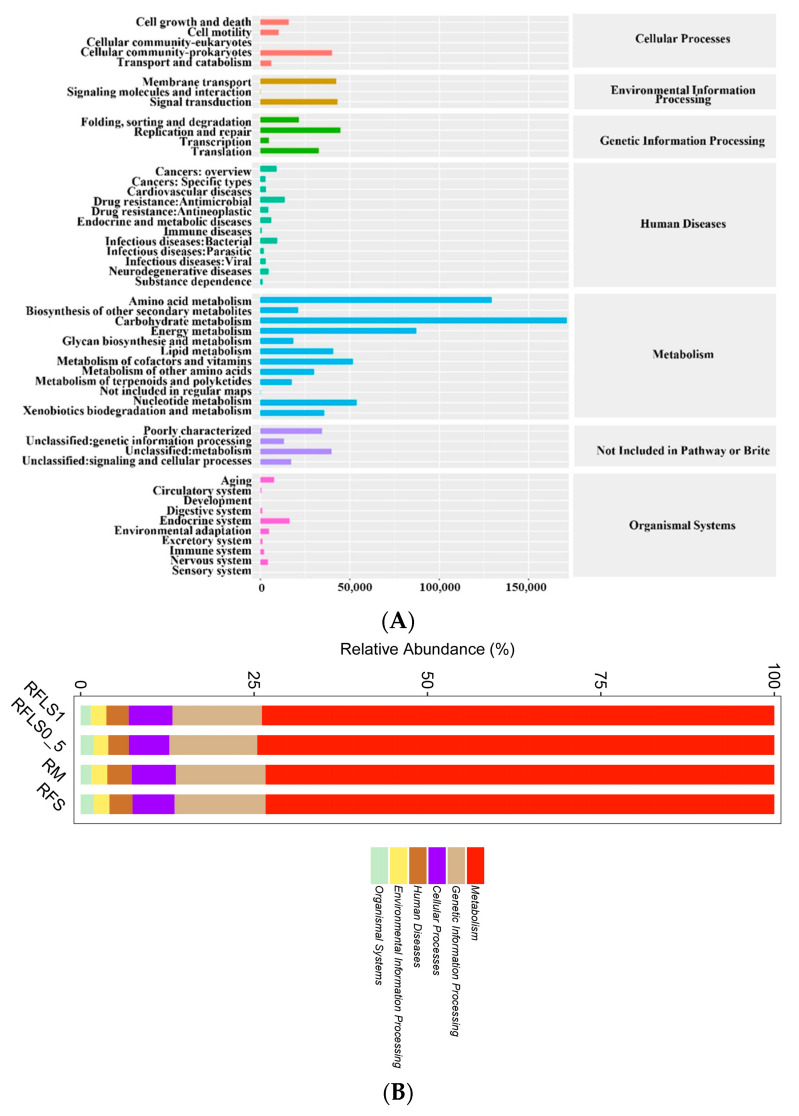

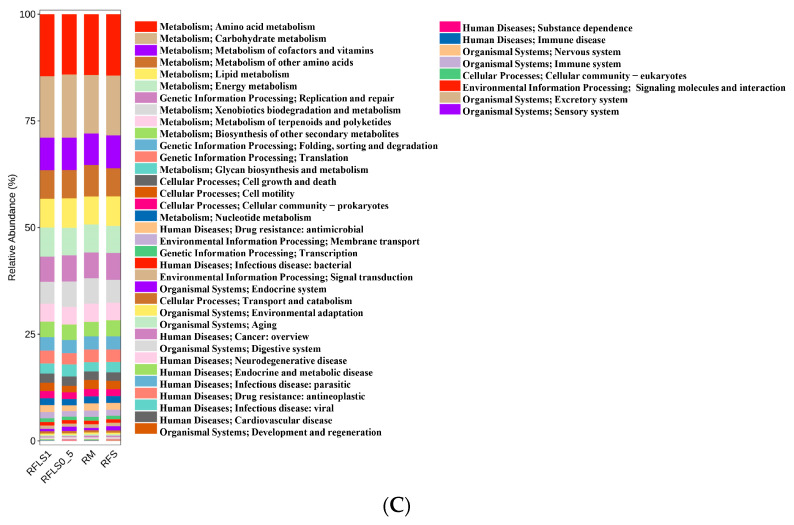
KEGG pathway analysis of soil metabolism. Note: (**A**) KEGG metabolic pathway annotation, (**B**) KEGG level 1 metabolic pathway classification, (**C**) KEGG level 2 metabolic pathway classification.

**Figure 6:**
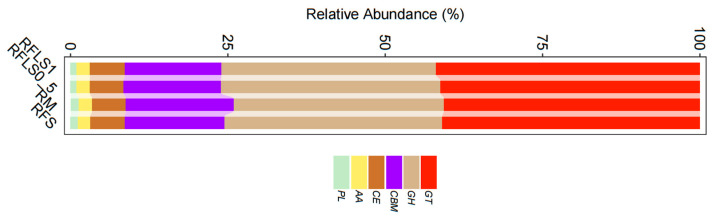
Analysis of soil CAZy database.

**Figure 7:**
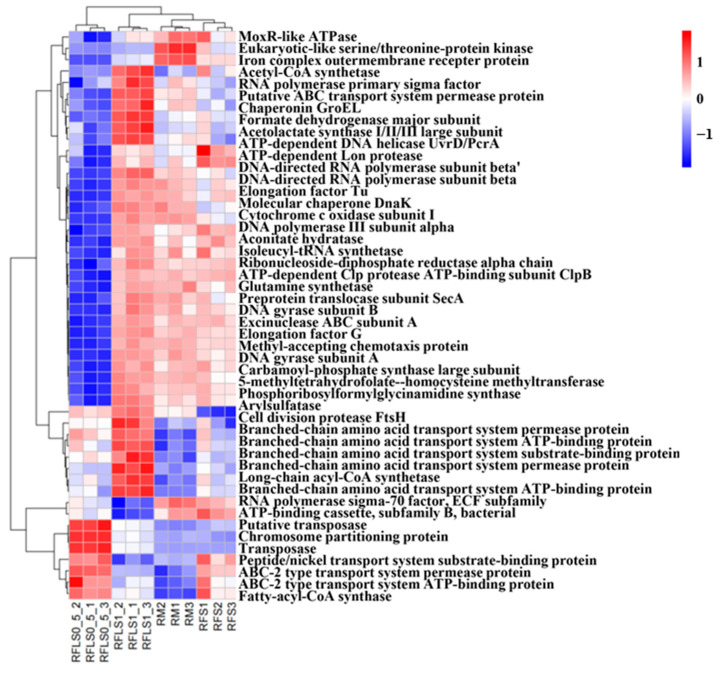
Clustering heatmap analysis of the top 50 differential KO functional units in soil.

As shown in Figure 5, optimal-density loach treatment significantly enhanced soil microbial metabolic functions. Metabolic functions dominated across all treatment groups (>70%), reaching 73.87 ± 0.13% in the RFLS 1 group and 74.55 ± 0.04% in the RFLS 0.5 group (Figure 5B, Table S1). Within secondary categories, the RFLS 1 group demonstrated the highest activities in amino acid metabolism (14.52 ± 0.09%) and carbohydrate metabolism (14.44 ± 0.06%) (Figure 5C, Table S2). Glycosyltransferases (GT) were the most abundant, with RFLS 1 group showing a higher proportion of GT abundance (41.93 ± 0.02%) than other groups (Figure 6, Table S3). Figure 7 reveals significant up-regulation in multiple key metabolic pathways in the optimal-density loach treatment group (RFLS 1). Specifically, enzymes related to nitrogen metabolism (e.g., glutamine synthetase and nitrate reductase), iron transport (e.g., ABC transport system), carbon metabolism (e.g., glucose kinase and isocitrate dehydrogenase), carbon dioxide fixation (e.g., carbon dioxide assimilation enzyme), and redox metabolism (e.g., cytochrome c oxidase) were significantly enriched in the RFLS 1 group.

## 4. Discussion

An appropriate density of co-cultured organisms raises efficiency of aquatic animals in utilizing feed resources, their metabolic rate, and energy storage, thus boosting their weight gain and nutrient accumulation [22,23]. Conversely, a high-density co-cultured system brings about intense interspecific competition and elevated stress responses. In this scenario, aquatic animals’ metabolic efficiency, nutrient absorption, and accumulation experience a dramatical decline, compromising both yields and economic benefits [23,24,25,26]. This aligns with our findings that moderate co-culture density optimizes growth conditions. In this study, frog growth was optimal at the appropriate loach density (RFLS 1 group) and declined at higher densities (RFLS 2 and RFLS 2.5 groups). Previous studies have indicated that low-density benthic animal activities in rice–fish or rice–crustacean co-culture systems significantly increase the relative abundance of *Actinobacteria* during the middle and early stages, thereby boosting soil organic matter decomposition potential [27]. As key decomposers of complex organic matter and producers of antimicrobial substances, the increased abundance of *actinobacteria* might have improved soil fertility and suppressed potential pathogen growth, consistent with functional enhancement effects observed in other rice–fish ecosystems [28,29,30]. Our research found that *Actinobacteriota* showed increased abundance in loach-treated groups, particularly in the RFLS 0.5 group, suggesting that loach activities, through stirring behavior, might have enhanced organic matter decomposition and provided more carbon sources for *actinobacteria*. *Proteobacteria* plays a key role in nitrogen fixing and carbon cycling [31]. Our study also revealed that *Proteobacteria* consistently maintained its dominance (>18%) across all treatment groups, indicating that its central role in paddy nitrogen cycling and carbon metabolism is minimally affected by loach activities, thereby ensuring the stability of key ecological functions. Similar observations have been reported in multiple studies, which highlighted that *Proteobacteria* demonstrates strong ecological adaptability to environmental perturbations across diverse integrated farming systems and acts as a “keystone supporting group” for functional stability in paddy systems [28,29,32].

Moderate animal disturbance enhances microhabitat heterogeneity, thereby boosting microbial diversity and metabolic functioning in paddy soils [33,34]. A previous study revealed that *Usitatibacter rugosus* may serve as microbial markers in metal-contaminated environments [35]. Moreover, *Pseudomonas aeruginosa* is an opportunistic pathogen with a high capacity to adapt to different factors [36]. In the present study, optimal-density loach treatment (RFLS 1) significantly inhibited the abundance of *Usitatibacter rugosus*, while suppressing proliferation of the potential pathogen *Pseudomonas aeruginosa*. Similar findings have been reported in rice–shrimp and rice–eel systems, suggesting that optimal-density animal disturbance is able to regulate the selective enrichment of microbial communities by altering soil physicochemical properties and nutrient distribution [37,38,39]. Additionally, optimal-density aquatic animal co-culture systems showed remarkable increases in microbial carbon source utilization and organic matter degradation rates [40,41,42]. In the present study, the optimal-density loach group (RFLS 1) shows high enrichment in amino acid metabolism (14.52 ± 0.09%) and carbohydrate metabolism (14.44 ± 0.06%). Moreover, glycosyltransferase (GT) (41.93 ± 0.02%) abundance significantly increased in the RFLS 1 group. These findings suggested that optimal-density loach treatment enhances microbe-mediated carbon and nitrogen cycling, providing more available nutrients for paddy ecosystems.

Glutamine synthetase is involved in ammonia fixation and nitrogen assimilation, while nitrate reductase catalyzes the conversion of nitrate to nitrite and then to ammonia. Their synergistic action enhances nitrogen availability. Analysis of the top 50 differential KO functional units in the RFLS 1 group showed evident enrichment of nitrogen metabolism-related enzymes (glutamine synthetase and nitrate reductase). In rice–fish co-culture systems, high-density fish activities significantly up-regulate nitrogen cycle-related gene expression, such as increased abundance of nifH, nosZ, and nirK genes [43,44]. Additionally, functions related to iron metabolism (ABC transport system), carbon metabolism (glucose kinase and isocitrate dehydrogenase), and redox metabolism (cytochrome c oxidase) were significantly up-regulated in the RFLS 1 group. Previous studies showed that fish activities effectively enhance the dynamic cycling of soil organic matter, Fe^2+^, and energy substrates; optimize the rhizosphere microenvironment; and boost rice photosynthetic efficiency and microbial metabolic activity [45,46]. Therefore, our findings implied that optimal-density loach treatment (RFLS 1) significantly enhances paddy soil ecological functions by promoting the growth of key microbial communities involved in nitrogen fixation and carbon metabolism. This regulatory mechanism provides a microbiological basis for optimizing rice–frog–loach integrated farming systems.

## 5. Conclusions

In conclusion, *P. nigromaculatus* in moderate-density loach treatment groups (10,000 individuals/667 m^2^, RFLS 1 group) demonstrated optimal weight gain within a rice–frog–loach integrated aquaculture system. At the soil level, there was significant enrichment of nitrogen-fixing bacteria of *Proteobacteria*, and suppression of the proliferation of the potential pathogen *Pseudomonas aeruginosa* and microbial markers in metal-contaminated environments of *Usitatibacter rugosus* in the RFLS 1 group. Additionally, moderate-density loach treatment significantly increased the activity of key metabolic pathways, including amino acid metabolism, carbohydrate metabolism, and nitrogen cycle-related enzyme systems, thereby improving the efficiency of carbon and nitrogen within a rice–frog–loach integrated aquaculture system.

## Figures and Tables

**Table 1:** Effects of loach density on growth performance of frogs.

Parameters	^1^ WGR	^2^ HSI	^3^ VSI	^4^ LBR
RFS	548.96 ± 103.11 ^d^	3.00 ± 0.54 ^c^	14.3 ± 1.72 ^c^	36.57 ± 3.02 ^a^
RFLS 0.5	746.13 ± 104.52 ^bc^	4.90 ± 0.85 ^b^	15.82 ± 2.92 ^b^	34.63 ± 2.27 ^ab^
RFLS 1	903.49 ± 182.97 ^a^	6.13 ± 1.31 ^a^	17.72 ± 2.37 ^a^	32.89 ± 1.77 ^b^
RFLS 1.5	844.42 ± 157.96 ^ab^	5.95 ± 1.05 ^a^	17.48 ± 2.02 ^a^	33.83 ± 2.66 ^b^
RFLS 2	769.04 ± 145.81 ^bc^	5.23 ± 0.88 ^ab^	16.11 ± 2.12 ^ab^	33.8 ± 2.55 ^b^
RFLS 2.5	695.04 ± 145.78 ^c^	4.95 ± 1.09 ^b^	16.14 ± 1.88 ^ab^	33.09 ± 3.22 ^b^

Data are presented as mean ± standard error (mean ± S.E.). Different lowercase letters indicate significant differences among treatment groups at *p* < 0.05. ^1^ WGR, weight gain ratio = 100 × (Wf − Wi)/Wi; ^2^ HSI, hepatosomatic index = 100 × Wl/Wf; ^3^ VSI, viscerosomatic index = 100 × Wv/Wf; ^4^ LBR, leg-to-body ratio = 100 × Wh/Wf. In the equation, Wi and Wf denote the initial and final average body weights (g) of frogs at the experiment’s start and conclusion, respectively. Wv represents the visceral mass (g); Wl is the liver mass (g); and Wh is the hind leg mass (g).

## Data Availability

The data presented in this study are available on request from the corresponding author. The data are not publicly available due to privacy restrictions.

## Supplementary Materials

The following supporting information can be downloaded at https://www.mdpi.com/article/10.3390/microorganisms13081794/s1, Table S1. KEGG level 1 metabolic pathway classification. Table S2. KEGG level 2 metabolic pathway classification. Table S3. Analysis of relative abundance in glycosyltransferases.
