# Supplementary material for: Effects of Paramisgurnus dabryanus Density on the Growth Performance of Pelophylax nigromaculatus and the Soil Microbial Communities Within a Rice–Frog–Loach Integrated Aquaculture System
Source: Microorganisms. 2025 Jul 31;13(8):1794. doi: 10.3390/microorganisms13081794 (PMC12388627; doi:10.3390/microorganisms13081794)
Supplement: Supplementary file 1 [file microorganisms-13-01794-s001.zip › microorganisms-3729529-supplementary.pdf]

Supplemental tables

Table S1. KEGG level 1 metabolic pathway classification.

Parameters	Metabolism (%)
RFLS 1	73.87±0.13[ab]
RFLS 0.5	74.55±0.04[a]
RM	73.35±0.11[b]
RFS	73.34±0.38[b]

Table S2. KEGG level 2 metabolic pathway classification.

Parameters	Amino acid metabolism	Carbohydrate metabolism
RFLS 1	14.52±0.09[a]	14.44±0.06[b]
RFLS 0.5	14.13±0.04[b]	14.84±0.09[a]
RM	14.24±0.06[ab]	13.72±0.03[c]
RFS	14.39±0.09[ab]	13.99±0.05[c]

Table S3. Analysis of relative abundance in glycosyltransferases.

Parameters	Glycosyltransferases (%)
RFLS 1	41.93±0.02[a]
RFLS 0.5	41.24±0.08[b]
RM	40.67±0.22[c]
RFS	40.97±0.10[b]
